# Sonoanatomy of the Nasal Ala for Botulinum Neurotoxin Injection

**DOI:** 10.1111/jocd.70035

**Published:** 2025-03-28

**Authors:** Kyu‐Ho Yi, Soo‐Bin Kim, Hyewon Hu, Konstantin Frank, Hugues Cartier, Sebastien Garson, Hee‐Jin Kim

**Affiliations:** ^1^ Division in Anatomy and Developmental Biology, Department of Oral Biology, Human Identification Research Institute, BK21 FOUR Project Yonsei University College of Dentistry Seoul Korea; ^2^ You and I Clinic Seoul Korea; ^3^ Department of Oral Anatomy, Institute of Biomaterial Implant, College of Dentistry Wonkwang University Iksan Korea; ^4^ Department of Plastic, Hand and Reconstructive Surgery University Hospital Regensburg Regensburg Germany; ^5^ Centre Médical Saint Jean Arras France; ^6^ Cabinet Médical Senlis France

**Keywords:** aesthetics, botulinum toxin, dynamic facial expressions, facial muscles, facial rejuvenation, injection technique, muscle contraction, nasal ala region, nasal anatomy, ultrasonography

## Abstract

**Background:**

The nasal ala region significantly affects facial aesthetics and function. Botulinum toxin injections may enhance nasal appearance, but their precise impact on underlying muscles remains unexplored. Understanding the muscular anatomy and behavior in this area is crucial for optimizing toxin application and achieving desired outcomes.

**Objective:**

This study aimed to ultrasonographically assess and characterize the muscular architecture of the nasal ala region in 32 participants, with the primary objective of delineating the specific muscles involved in nasal aesthetics.

**Methods and Materials:**

This cross‐sectional study included 32 participants (15 females and 17 males, aged 20–65) with no history of nasal surgery or botulinum toxin injections in the nasal region. Ultrasonographic evaluation was performed to assess the superficial and deep muscular layers in the nasal ala region, using ultrasonography to visualize the targeted muscles and measure their depth.

**Results:**

Ultrasonographic analysis revealed distinct muscular structures in the nasal ala region among participants. Simultaneous movements of the associated muscles were observed, exhibiting diverse depths.

**Conclusion:**

Ultrasonographic evaluation in our study population elucidated the anatomical nuances of the underlying muscles involved in nasal anatomy. These findings establish a foundation for a more targeted and precise approach in administering botulinum toxin injections, potentially optimizing nasal aesthetics outcomes.

## 1 Introduction

Facial aesthetics has garnered significant attention in the field of cosmetic procedures, and botulinum toxin (BoNT) injections have emerged as versatile tools in this domain [1, 2, 3]. Traditionally recognized for their role in reducing facial wrinkles, BoNT injections are now also utilized to refine nasal regions [4].

**FIGURE 1:**
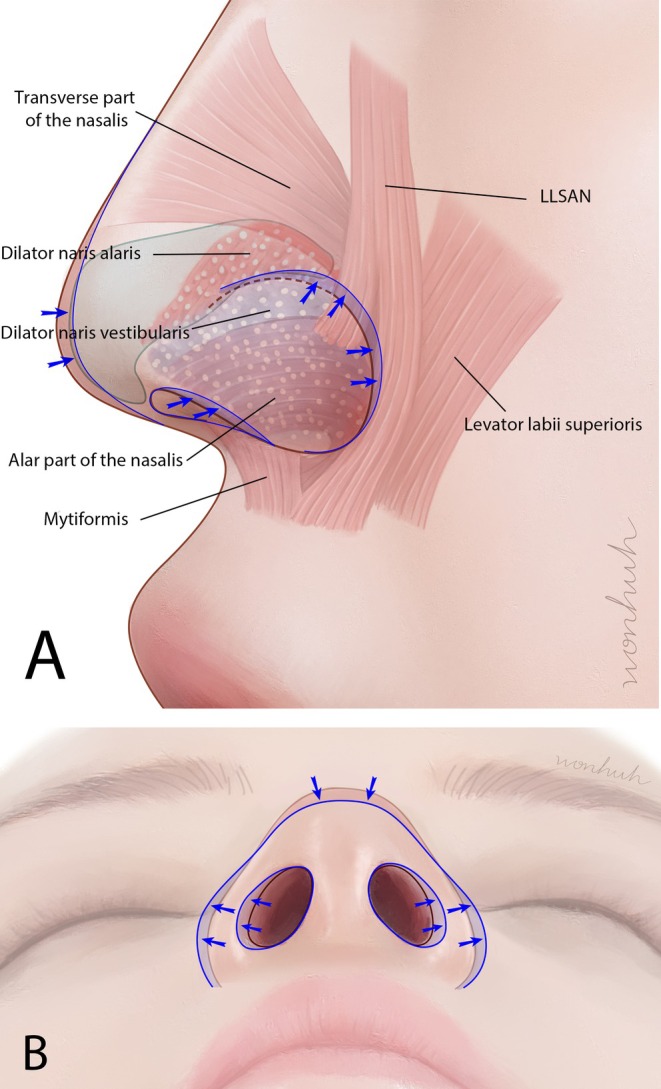
A schematic illustration showing the nasal alar‐associated muscles from lateral (A) and inferior (B) perspectives. The diagram identifies the contracted muscles, namely the myrtiformis, dilator naris anterior (DNA), dilator naris vestibularis (DNV), alar part of the nasalis, transverse part of the nasalis, and the levator labii superioris alaeque nasi (LLSAN). The figure A is redrawn from the literature of Hur et al. [7].

The injection of BoNTs into the nasal area has typically been limited to the treatment of nasal wrinkles [5]. However, recent practices have expanded to administering BoNT injections in the ala region [6]. Several muscles, including the myrtiformis, dilator naris anterior (DNA), dilator naris vestibularis (DNV), ala part of the nasalis, transverse part of the nasalis, and levator labii superioris alaque nasi (LLSAN), exist in this region. Contraction of these muscles induces movement in the ala area (Figure 1). By selectively inhibiting these actions, BoNT modulates involuntary muscle activity responsible for ala flaring and other dynamic movements. Moreover, the nasal ala region encompasses the lateral aspect of the nose, contributing significantly to the overall facial appearance and nasal contour.

However, administering BoNT injections for nasal ala muscle treatment can lead to complications, such as asymmetrical ala shape and paralysis of nearby muscles, including the orbicularis oris muscle. These complications often arise because of insufficient awareness of the anatomical factors associated with the procedure [8]. To avoid undesired effects, injections are administered precisely and directly into each delicate muscle based on sound anatomical knowledge.

In this study, we utilized ultrasonography to examine the anatomical structure and dynamic movements of the nasal ala, aiming to identify the most suitable landmarks for guiding BoNT administration. We also explored the intricacies of applying BoNT in the nasal ala region.

## 2 Materials and Methods

This cross‐sectional study included 32 Korean participants (15 females and 17 males) with a mean age of 37.4 years (range: 18–62 years). Individuals who had not undergone nasal interventions, nasal surgery, or BoNT injections into the nasal region were evaluated using ultrasonography. Written informed consent was obtained from all participants, and this study adhered to the principles of the Declaration of Helsinki. The research protocol received approval from the Institutional Review Board (IRB) of Yonsei University Dental Hospital (IRB No. 2‐2023‐0050; approval date: October 19, 2023).

The examination focused on assessing the superficial and deep muscular layers within the nasal ala region during resting and dynamic facial expressions. Ultrasonography was used to visualize the muscles associated with the ala, including the transverse part of the nasalis, ala part of the nasalis, DNV, DNA, LLSAN, and levator labii superioris (LLS).

Ultrasonographic images of these muscles were captured using a real‐time two‐dimensional B‐mode ultrasonography device equipped with a high‐frequency (18 MHz) linear transducer (Sonimage HS1; KONICA MINOLTA, Tokyo, Japan). Ultrasonographic imaging and depth measurements were performed by positioning the probe horizontally along an imaginary line connecting the pronasal area and the alar groove. The probe was also aligned horizontally along the lower border of the nose at the pronasal level and placed horizontally between the pronasal and subnasal areas to capture ultrasonographic images from these three regions.

To ensure the reliability of the ultrasonographic measurements, intra‐observer reliability was assessed by having the same observer repeat the measurements on 10 randomly selected participants after a 2‐week interval. Intraclass correlation coefficients (ICCs) were calculated, yielding a value of 0.95, indicating excellent consistency.

The normality of the data distribution was assessed using the Shapiro–Wilk test. Differences in the depth from the skin to the muscle between male and female groups were analyzed using the Mann–Whitney *U*‐test. A significance level of *p* < 0.05 was adopted for all analyses. Statistical analyses were conducted using SPSS software (version 23.0 for Windows, SPSS Inc., Chicago, IL, USA).

## 3 Results

**FIGURE 2:**
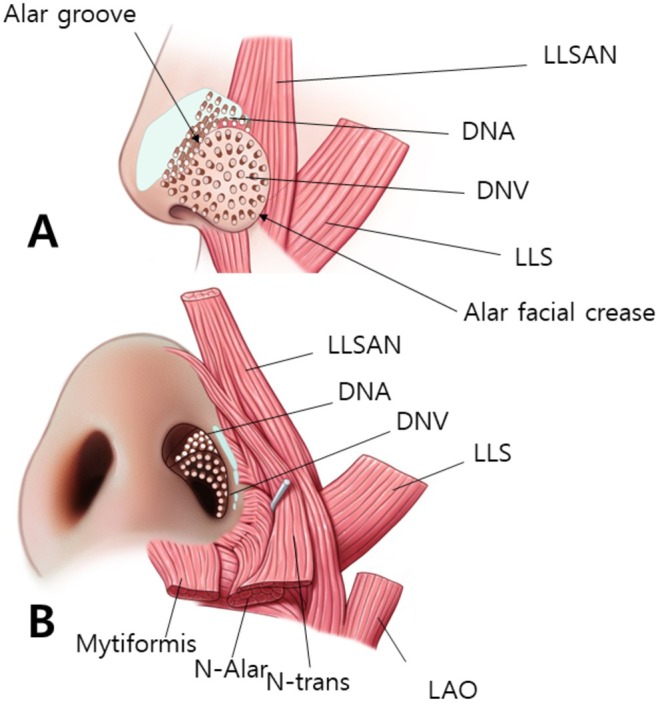
The figure displays schematic representations illustrating the nasal musculature from both lateral (A) and inferior (B) perspectives. In panel A, the lateral view delineates the arrangement of muscles within the nose. The dilator naris anterior (DNA) originates from the frontal surfaces of the lateral portion of the lateral crus and the accessory alar cartilage, attaching to the adjacent skin of the nose. Its lower insertion extends to the alar groove. The dilator naris vestibularis (DNV) is situated between the external and vestibular skin of the ala region, encompassing the dome‐shaped alar lobule. Panel B shows the inferior perspective of the nasal musculature. The ala part of the nasalis arises from the maxilla and connects to the alar facial crease and the deeper surface of the external ala skin. In addition, the diagram indicates the presence of other relevant muscles, such as the levator anguli oris (LAO), levator labii superioris (LLS), levator labii superioris alaeque nasi (LLSAN), and the transverse part of the nasalis, while representing the alar facial crease with a dotted line and alar groove with a straight line. The figure A and B is redrawn from the literature of Hur et al. [7].

The ala portion of the nasalis muscle blends with the DNV extending from the alar facial crease to the lower and lateral sections of the alar lobule. The muscular fibers of the ala portion of the nasalis insert toward the external skin, contributing to the downward pull and narrowing of the ala muscle. Notably, although the ala part of the nasalis does not completely envelope the entire ala section, it exhibits greater thickness in the lateral section of the lobule, where it converges with the LLSAN and LLS muscles (Figure 2A).

**FIGURE 3:**
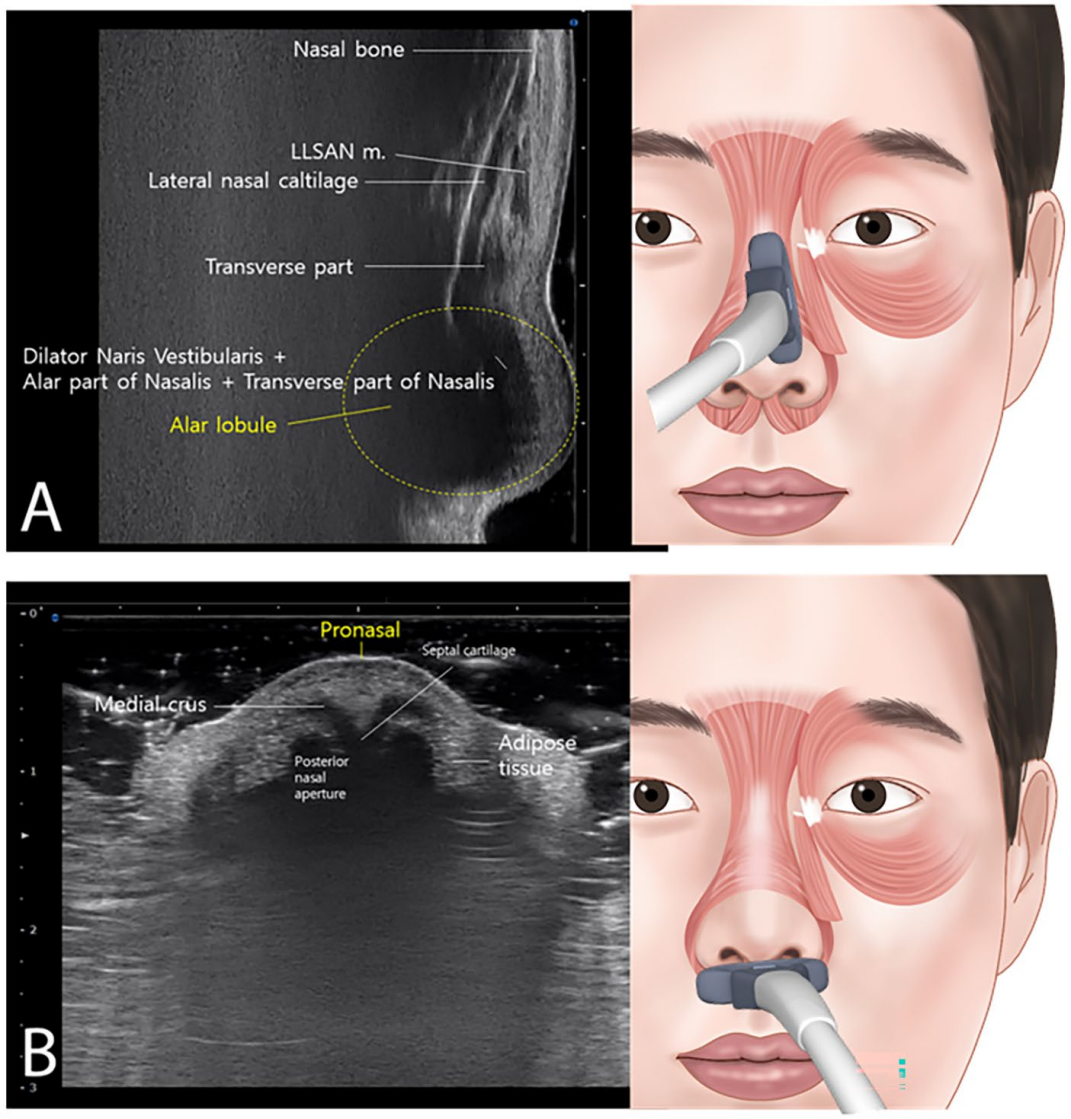
Ultrasound observations during probe placement in specific nasal areas. In panel A, the probe placed diagonally and laterally for observing the transverse part of the nasalis reveals a convergence of the dilator naris vestibularis (DNV), ala part of nasalis, and transverse part of nasalis at the lateral section of the alar lobule. The resulting hypoechoic image displays the presence of the lateral nasal cartilage and associated muscles. In panel B, the probe positioned on the pronasal area provides a view of the medial crus along with the septal cartilage, depicted in the hypoechoic image (B mode, 18‐MHz linear transducer).

The DNA is situated above the alar groove and DNV is located below the alar groove. The myrtiformis, consisting of the depressor septi nasi and depressor alae nasi muscles, was observed at the base of the nasal opening (Figure 2B). When the probe was positioned along the lateral border of the nose to observe the transverse part of the nasalis (Figure 3A), the DNV, along with the ala and transverse parts of the nasalis were found to merge at the lateral segment of the alar lobule. Hypoechoic imaging revealed the presence of the lateral nasal cartilage and associated muscles. Additionally, placing the probe horizontally between the pronasal and subnasal areas revealed the medial crus and septal cartilage as hypoechoic images (Figure 3B).

**FIGURE 4:**
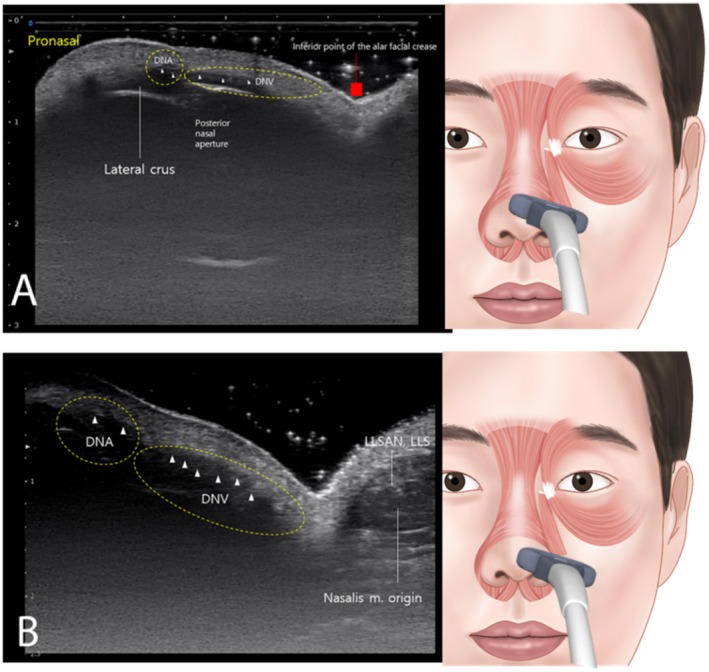
The ultrasound observations obtained through probe placement in specific nasal regions. In panel A, placing the probe diagonally at the pronasal area shows the presence of the lateral crus and the dilator naris anterior (DNA), and dilator naris vestibularis (DNV), originating from the posterior nasal aperture and extending vertically to the dermal layer. Additionally, the alar groove acts as a demarcation line separating the DNA and DNV. Panel B displays that the nasalis muscle, consisting of the ala and the transverse parts, originates from the maxilla and intertwines with the levator labii superioris alaeque nasi (LLSAN) and levator labii superioris (LLS) (B mode, 18‐MHz linear transducer).

Positioning the probe horizontally along an imaginary line connecting the pronasal area and the alar groove revealed the DNA and DNV originating from the posterior nasal aperture and traversing vertically to the dermal layer, with the alar groove acting as a demarcation between the two (Figure 4A). At the pronasal area level, when a probe was placed horizontally along the lower border of the nose, it showed that the alar and transverse parts of the nasalis muscle originate from the maxilla and intermingle with the LLSAN and LLS (Figure 4B). Moreover, when flaring the nose outward, simultaneous distinct movements of the nasal musculature were observed, as shown in Video S1.

The depth from the skin to the muscle was measured as 1.94 ± 0.17 mm horizontally along an imaginary line connecting the pronasal area and the alar groove, 2.85 ± 0.04 mm at the pronasal area level with the probe placed horizontally along the lower border of the nose, and 4.55 ± 0.06 mm horizontally between the pronasal and subnasal areas. Ultrasonographic images were consistent across all 32 participants, with no observable discrepancies. Additionally, no significant differences were found between male and female groups (*p* > 0.05).

## 4 Discussion

In this cross‐sectional study, we found that ultrasonography effectively identifies suitable landmarks for BoNT administration in the nasal ala region, leading to refined nasal contours and minimized nostril flaring. These findings suggest that precise anatomical knowledge significantly enhances the efficacy and safety of BoNT injections, reducing the risk of complications such as asymmetrical ala shape and muscle paralysis.

**FIGURE 5:**
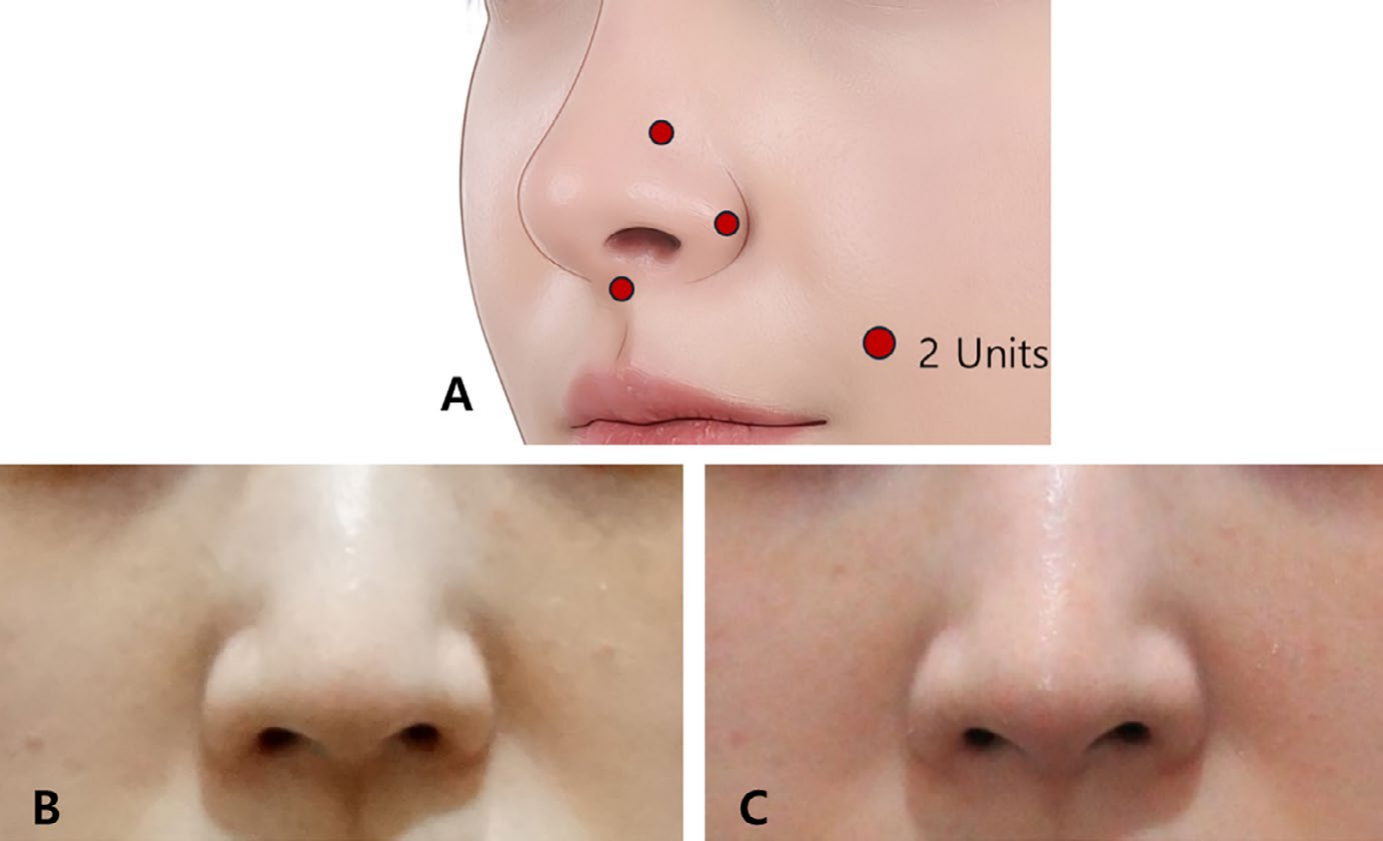
By targeting the actions of these muscles, one can reduce sharp and thick alar movements, prevent nasal tip depression, and minimize alar widening through a precise injection strategy that focuses on the transverse part of the nasalis, dilator naris anterior, alar part of the nasalis, dilator naris vestibularis, levator labii superioris alaeque nasi, and myrtiformis (A). The three‐point injection for nasal shaping has been conducted at locations above the alar groove. A 35‐year‐old female with a widened alar received a single treatment guided by the above injection points. Before the treatment (B) and 2 months after the treatment (C).

Strategic targeting of muscles such as the transverse part of the nasalis, DNA, and LLSAN can reduce sharp alar movements, prevent nasal tip depression, and minimize alar widening. Table 1 provides a detailed overview of these muscles, outlining their roles and the expected effects when targeted. Based on the authors' guidelines, key injection points include the alar groove, the lateral inferior part of the alar lobule, and the nasal spine. This approach primarily targets muscles such as the transverse part of the nasalis, DNV, and the nasal insertion of the LLSAN, contributing to more defined nasal contours by minimizing alar widening and nasal tip depression. Additionally, an injection at the nasal spine can paralyze the myrtiformis, further refining the nasal shape (Figure 5). Recognizing nasal anatomy is also critical when integrating BoNT with surgeries like rhinoplasty, ensuring a harmonious transition between the nasal base and upper lip [6].

**TABLE 1:** Muscles of the nasal alar region, functions, expected outcomes upon botulinum toxin injection.

	Function	Expected outcome upon injecting
Dilator naris anterior (DNA)	Lateral crus shape, alar groove formation, widen nasal passages	Define lateral alar groove, prevent nostril flaring, induce nasal sidewall thinning
Dilator naris vestibularis (DNV)	Widens posterior nasal aperture, lateral ala movement during flaring, widen nasal passages	Prevent nostril flaring, induce nasal sidewall thinning
Ala part of the nasalis	Influence nose width during downward and medial elongation	Prevent upward alar elevation, inhibit lateral widening, prevent nose tip plunging, avoid droopy nose appearance
Levator labii superioris alaque nasi (LLSAN)	Pull nose superolaterally, create lateral alar groove	

Hur et al. [7] highlighted the structure and positioning of the DNV muscle, which lies between the vestibular and external ala skin. This muscle, along with the DNA and alar nasalis, contributes to nasal flaring and vestibular movements. Together, these muscles play a critical role in shaping the nasal ala and are consistent with the ultrasonographic findings of this study.

Moreover, in this study, ultrasonography was used to measure the thickness of the injection sites, enabling the proposal of effective injection depths. The distance from the skin to the muscle was 1.94 ± 0.17 mm at the alar groove, 2.84 ± 0.04 mm at the lower border of the nose, and 4.55 ± 0.06 mm at the nasal spine. Based on these findings, and considering both maximum and minimum values, an injection depth of about 1.9 mm at the alar groove, about 2.8 mm at the lower border of the nose, and about 4.5 mm at the nasal spine is suggested to accurately target the muscles in the nasal ala region.

Yi et al. [9] emphasized the importance of precise BoNT injection sites and external anatomical landmarks, proposing a framework for improving outcomes and minimizing complications such as nasal deviations or filler migration. Injecting BoNT into the alar part of the nasalis and LLSAN has been shown to prevent nostril flaring and lateral widening while maintaining nasal tip stability. Patients with widened nasal openings particularly benefit from targeting the ala facial crease, with injection adjustments made for individual anatomical variations. This tailored approach highlights the potential of BoNT in both aesthetic and functional refinements of the nasal region.

The primary limitation of this study is its small sample size (32 participants), which limits the generalizability of the findings. Additionally, while our intra‐observer reliability assessment indicated excellent consistency (ICC = 0.95), the study was conducted using a single imaging modality. Future research with larger cohorts and diverse imaging modalities is needed to validate and expand upon these results. A thorough understanding of nasal anatomy through ultrasonography is essential for achieving successful BoNT injections in the nasal ala region, ensuring optimal outcomes.

## Author Contributions

All authors have reviewed and approved the article for submission. **Kyu‐Ho Yi, Soo‐Bin Kim, Hee‐Jin Kim:** conceptualization. **Kyu‐Ho Yi, Soo‐Bin Kim; Hugues Cartier:** writing – original draft preparation. **Kyu‐Ho Yi, Soo‐Bin Kim, Sebastien Garson, Frank Konstantin:** writing – review and editing. **Hyewon Hu, Soo‐Bin Kim:** visualization. **Hee‐Jin Kim:** supervision.

## Conflicts of Interest

The authors declare no conflicts of interest.

## Supporting information


**Video S1.** Noticeable movements of the muscles in the nose are observed.

## Data Availability

The data that support the findings of this study are available from the corresponding author upon reasonable request.
