# Correction to “Sonoanatomy of the Nasal Ala for BotulinumNeurotoxin Injection”

**DOI:** 10.1111/jocd.70429

**Published:** 2025-08-29

## Abstract

YiK.‐H., , KimS.‐B., , HuH., , FrankK., , CartierH., , GarsonS., and KimH.‐J., (2025). Sonoanatomy of the Nasal Ala for Botulinum Neurotoxin Injection. J Cosmet Dermatol, 24: e70035. 10.1111/jocd.70035.40152086 PMC11951073

The affiliation of the corresponding author, Hee‐Jin Kim, was incorrectly listed as 2 (You and I Clinic, Seoul, Korea). It should be corrected to 1 (Division in Anatomy and Developmental Biology, Department of Oral Biology, Human Identification Research Institute, BK21 FOUR Project, Yonsei University College of Dentistry, Seoul, Korea).

We apologize for this error.

Yi, K.‐H. , Kim, S.‐B. , Hu, H. , Frank, K. , Cartier, H. , Garson, S. and Kim, H.‐J. (2025). Sonoanatomy of the Nasal Ala for Botulinum Neurotoxin Injection. J Cosmet Dermatol, 24: e70035. 10.1111/jocd.70035.40152086 PMC11951073

The affiliation of the corresponding author, Hee‐Jin Kim, was incorrectly listed as 2 (You and I Clinic, Seoul, Korea). It should be corrected to 1 (Division in Anatomy and Developmental Biology, Department of Oral Biology, Human Identification Research Institute, BK21 FOUR Project, Yonsei University College of Dentistry, Seoul, Korea).

We apologize for this error.

