# Correction to “Sonoanatomy of the Nasal Ala for Botulinum Neurotoxin Injection”

**DOI:** 10.1111/jocd.70588

**Published:** 2025-12-05

K. H. Yi, S. B. Kim, H. Hu, K. Frank, H. Cartier, S. Garson, and H. J. Kim, “Sonoanatomy of the Nasal Ala for Botulinum Neurotoxin Injection,” Journal of Cosmetic Dermatology 24, no. 4 (2025): e70035. https://doi.org/10.1111/jocd.70035.

In the published version of the article, the funding year is stated inconsistently.

The first page correctly lists the support year as 2025, whereas the Acknowledgment section incorrectly states it as 2024.

We apologize for this error.

